# Developing a mechanism of construction project manager’s emotional intelligence on project success: A grounded theory research based in China

**DOI:** 10.3389/fpsyg.2022.693516

**Published:** 2022-09-26

**Authors:** Qi Zhang, Shengyue Hao

**Affiliations:** ^1^School of Management, Zhejiang University, Hangzhou, China; ^2^Network Security and Informationization Research Institute, Huaxin Consulting Co., Ltd., Hangzhou, China; ^3^Department of Engineering and Management, School of Economics and Management, Beijing Jiaotong University, Beijing, China

**Keywords:** emotional intelligence (EI), construction project managers (CPMs), project success, grounded theory (GT), mechanism building

## Abstract

A project manager’s emotional intelligence (EI) is essential to project success. However, the mechanism in this cause and effect remains a black box in extant literature. China is now the world’s largest construction market, and figuring out the mechanism of construction project manager’s (CPM’s) EI on project success is meaningful for developing the global construction market. This study conducted an in-depth interview with 24 CPMs with more than 5-year experience in construction project management. The grounded theory was employed to profile the application of CPM’s EI and to build the multilevel mechanism that explains the influence of CPM’s EI on project success. The mechanism framework conforms to the existed input–process–output (IPO) theory. It consists of a team-level mechanism (including the positive team atmosphere, shared vision, and team cohesion) and an individual-level mechanism (i.e., organizational citizenship behavior directed at the organization, perceived supervisor support, trust in leader, and subordinate’s psychological and emotional health). This study further proposed that the effect of this mechanism does not work immediately but develops with time passing. Implications for further research and project management practice are discussed in the end.

## Introduction

With the rapid development of China’s economy and society, in the past few decades, financial funds have been adequately flowing to the construction industry (China’s pillar industry), and an increasing number of construction projects have been undertaken. China is now the world’s largest construction market and is expected to maintain this position in the near future (Yan et al., 2019). Under the background of the “Belt and Road Initiative,” Chinese overseas investment in infrastructure promoted the internationalization process of Chinese construction enterprises. The construction industry has been perennially considered as one of the most complicated contexts to lead people effectively to improve work output and achieve success (Potter et al., 2018). Besides, due to the complexity, uncertainty, ambiguity, temporary, and fragmentation nature of the new era’s construction project (Stark et al., 2014; Ksia̧żek et al., 2015; Walker et al., 2017), the achievement of project success continues to be a challenging goal and has caused growing concern for construction companies. Considering the unique position of the Chinese construction market and the potentially massive impact on infrastructure construction in countries along the Belt and Road, successful project outcomes of Chinese construction companies could significantly contribute to the global construction industry.

A construction project is a people business (Skipper and Bell, 2006), and it is widely accepted that the “people” factor now plays a crucial role in almost any type of project (Dillon and Taylor, 2015; Sundqvist, 2019). As construction project management practice and research fields have evolved, the heightened expectations of project managers and their leadership are becoming more evident (Sundqvist, 2019). Some argued that the project manager’s leadership might be the most critical factor of successful project outcomes in the construction industry (Sunindijo et al., 2007; Rezvani et al., 2016). In developing our research interests, we noticed that the role of emotion or emotional intelligence (EI) is becoming one of the most topical areas in organizational-level research. CPM’s EI is now increasingly investigated and emphasized as an essential determinant in how successful leaders manage in the workplace (Rezvani et al., 2016). Salovey et al. (2003) defined EI as the ability to perceive and express emotions, understand and use them, and manage them to foster personal growth. With the unexpected popularity of the book *Emotional Intelligence* written by Daniel Goleman in China, the EI concept has become popular and has drawn wide attention in Chinese society and organizations in the recent two decades. This situation provides a rich foundation to explore CPM’s EI in the Chinese context.

Based on EI theory and its research findings in the psychology field, management scholars and practitioners viewed EI leader as one type of person-focused leadership (Booms et al., 2016) and pointed that EI may hold the key to improving project manager’s career success as a leader and the performance of project team (Turner and Müller, 2005). Individuals with high EI are able to make informed decisions, better cope with environmental demands and pressures, effectively handle conflict, communicate in exciting and assertive ways, and make others feel better in their work environment (Love et al., 2011). For CPMs who are constantly confronted with communication issues and complex relationships, it seems that they should embrace this concept and use it in project management for better performance.

However, although some scholars found a positive association between CPM’s EI and project outcomes (e.g., Maqbool et al., 2017), construction practice seems to hold an uncertain attitude toward EI to some extent. For example, based on interview research from the UK construction industry, Lindebaum and Cassell (2012) found that CPMs avoided EI under the male-dominated culture. This view, though, comes from a minority, which draws our attention since Chinese construction is also a historically male-dominant industry (Zhang and Zhao, 2015), and most employees in the industry are male (Swider, 2016). Therefore, we are automatically interested in the view of Chinese CPMs and whether they also avoid using EI in project management under a similar male-dominated culture. On the other hand, except for the male-dominated culture, the Chinese construction industry roots in Chinese traditional guanxi culture, the informal connections bound by implicit psychological contracts to exchange reciprocity, nurture mutual commitment, and aim for long-term relationships (Ren and Chadee, 2017). Some dimensions of EI (such as using emotion to facilitate thought) meet the social and communication demands of guanxi culture. As managerial styles do vary from culture to culture (Tang et al., 2010), the above phenomenon based on the developed country does not seem to be adaptive in the Chinese situation. Therefore, the first question we are interested in is:

*RQ1.* How do Chinese CPMs view EI (do they avoid using EI), and what are the typical scenarios they use EI skills for project management?

On the other hand, we found that most existing literature tends to investigate the direct relationship between CPM’s EI and project outcomes (Zhang and Fan, 2013; Maqbool et al., 2017). Besides, although some discrete studies explored a few mediating factors in this relationship, such as transformational leadership (Zhang et al., 2018), the mechanism between CPM’s EI and project success still lacks systematic discussion and remains a black box. Therefore, the second research question of this study is:

*RQ2*. What is the mechanism of CPM’s EI impacts on project success?

Solving the above-proposed questions is a sense-making and theory-building process. Qualitative research emphasizes the meaning rather than the measurement of phenomenon (Andersen and Kragh, 2010) and meets the requirements of the current paper. As such, we adopted a combination of interview research and grounded theory methodology (GTM) to depict the practice of CPM’s EI and investigate the mechanism for CPMs using EI to achieve project success. By employing in-depth interviews and GTM, this paper built a framework that includes team-level and individual-level paths.

## Literature review

### Emotional intelligence

To date, there is no unified operational definition of EI. Salovey and Mayer’s (1990) definition (which is often referred to as “ability EI”) mentioned in the Introduction is skill-based and focuses on cognitive aptitude, similar to a traditional intelligence—IQ, which emphasizes the procedure of emotional information (Day and Carroll, 2004; Davis, 2011). Some other scholars considered EI in a more mixed perspective. They expanded ability EI and argued that EI includes a set of behaviors, characters, traits, skills, and competencies (Kerr et al., 2013). For example, Bar-On (2006) defined EI as a cross section of interrelated emotional and social competencies, skills, and facilitators. The current paper adopted ability EI as the theoretical basis, since the latter view or the “mixed EI” is frequently and justifiably criticized for the lack of theoretical clarity (Hughes and Evans, 2018). Indeed, when a structure is so broad that it can reasonably accommodate nearly everything, it is so changeable in nature and therefore meaningless (Hughes and Evans, 2018). The adoption of ability EI helps this study to establish a relatively purer view on CPMs’ leadership. On the other hand, a previous case study conducted in developed countries (Lindebaum and Cassell, 2012) also adopted the ability EI framework. The adoption of ability EI leaves space for the comparison of the findings of this paper with previous research. Salovey and Mayer (2003) constructed ability EI from four dimensions or branches: perceiving emotion (Branch 1), using emotion to facilitate thought (Branch 2), understanding emotion (Branch 3), and managing emotion (Branch 4). Branch 1 concerns the ability to identify emotions in oneself and others. Branch 2 means the ability to generate, use, and feel the emotion as necessary to communicate feelings. Branch 3 refers to the ability to comprehend emotional information. Branch 4 is defined as the ability to be open to feelings, to regulate them in oneself and others to promote personal understanding and growth.

### Construction project success

Success is always the ultimate goal of project management (Luo et al., 2017), and project success relies on the nature of the project (Negash and Hassan, 2020). For construction projects, traditional project performance was estimated by the time/cost/quality (which refers to the iron triangle standard) (Pollack et al., 2018) or financial aspects, such as investment and profit per unit (Besharov et al., 2016). Compared to project performance, the “project success” concept focuses more on “soft” and “people” issues, such as team-related criteria (e.g., team cooperation) and external stakeholder’s satisfaction (Albert et al., 2017). Project success emphasizes organizational long-term benefits and opportunities generated by the project (Ahmed et al., 2016). Prior literature implies that building a project success criteria system is more practical than clarifying one uniform definition. For example, Sadeh et al. (2000) divided the success dimension into meeting design goals, the benefit to the end user, the benefit to the developing organization, and the benefit to the defense and national infrastructure. Chan et al. (2004) proposed the project success standard system based on the literature review, i.e., time, cost, quality, health and safety, environmental performance, participants’ satisfaction, user satisfaction, and commercial value. In conclusion, the concept of construction project success is comprehensive and can involve various project participants and other stakeholders related to the project. We adopted the success standard Chan et al. (2004) proposed because of the number of citations and the solid literature review of this study.

### Construction project manager’s emotional intelligence and construction project success

Some prior quantitative studies explored the direct influence of CPM’s EI on project success, such as Zhang and Fan (2013) and Sang et al. (2018). However, although CPM’s EI has been argued to resolve project management issues and positively impact project success, the underlying mechanisms influencing the EI–project success relationship remain unknown. As Müller and Jugdev (2012) suggested, scholars need to explore variables that potentially mediate project manager characteristics (such as EI) and project success. We attempt to find these potential variables and build the underlying mechanisms influencing the EI–project success relationship.

Besides, a few studies tried to explain the impact of CPMs’ EI on project success by leadership, the process of influencing subordinates to facilitate relevant organizational goals attainment (Kasapoğlu, 2014). Leaders are identified as one specific type because of the qualities or characteristics they possess. Taking transformational leadership as an example, transformational leaders usually use their charming personalities to foster a collective sense of mission and inspire their subordinates to provide intellectual challenges (Maqbool et al., 2017; Zhang et al., 2018). Salovey and Mayer (1990) suggested behavioral manifestations of emotionally intelligent individuals include individualized consideration, empathy, and respect. These behavioral manifestations are transformational in nature (Sitter, 2004). In other words, some behavioral presentations of EI leaders are similar to transformational leadership. Therefore, we believe that using the leadership perspective to explain the EI–project success relationship’s mechanism is not powerful enough. Because both EI and leadership are characteristics or attributes that belong to CPM themselves, some other potential mediation roles are still not revealed.

On the other hand, although mainstream result supports a positive relationship between CPM’s EI and project success, we also noticed that a few studies based on developed country context such as the UK (e.g., Lindebaum and Cassell, 2012; Lindebaum and Jordan, 2012) proposed some different opinions. For example, Lindebaum and Cassell (2012) concluded that emotions were unnecessary and inappropriate in UK construction project workplaces. However, guanxi activities are deeply rooted in Chinese culture and play a vital part in Chinese organizations (Zhan et al., 2018), and the implementation of guanxi activities was directly influenced by emotions (Barbalet, 2018). Therefore, the phenomenon of avoiding EI by CPM theoretically does not adapt to China’s construction practice. Besides, Lindebaum and Cassell (2012) proposed the representative views of the avoidance of EI mentioned above 8 years ago, and construction project nature is changing in these years. To figure out whether the utility of EI is culture-specific or period-specific, our focus here is on the Chinese construction project context in the current days.

## Methodology

### Grounded theory methodology and in-depth interview

The current study employs a combination of GTM and in-depth interviews. GTM closely links empirical research with theory construction and provides detailed procedures to summarize and develop concepts or theories through systematic analysis of empirical data and *ground* in it (Glaser and Strauss, 2017). There are two distinct versions of GTM: Glaser’s version of grounded theory (or classic GT) and Strauss’s version of GT (Glaser and Holton, 2004). According to Walker and Myrick (2006), many of the differences between Glaser’s and Strauss’s versions lie in their perspectives regarding the data analysis process, precisely the procedures they used. Glaser’s GT divides the coding process into two stages: substantive and theoretical coding. Substantive coding concerns building categories and their definition, characters, and properties, and this process consist of two subphases, open and selective coding. The theoretical coding weaves the substantive codes together into hypothesis and theory, and it occurs at the conceptual level (Walker and Myrick, 2006). On the contrary, Strauss’s version offers a somewhat prescriptive approach and uses literature and predetermined coding schema, and they divided the coding process into three phases: open, axial, and selective coding (Strauss and Corbin, 2014). Since we intend to build a mechanism that did not appear before and immerse ourselves into interview data as more as possible, the foundation of the current work is much closer to Glaser’s version of GT, and we employed the two-process coding of Glaser’s GT too.

**FIGURE 1:**
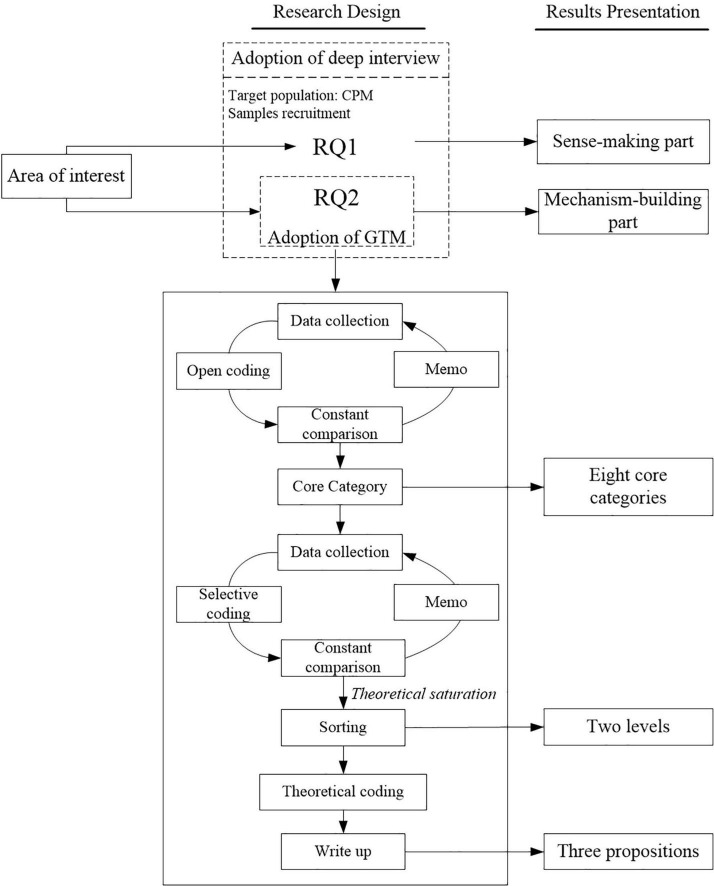
Research design of the current study.

An in-depth interview is a crucial component in most qualitative research, through which researchers can understand the life and working experience of a group, explore the formation process of specific social phenomenon, and put forward ideas and methods to solve problems. It is also a key component of data collection in GTM (Lakshman, 2007). By combining the above two methods, the authors accumulated a large amount of interview data to build the mechanism of CPM’s EI on project success. The research design of this study is shown in Figure 1.

### Samples

Extant literature often employs snowball sampling, quota sampling, purposive sampling, or heterogeneous sampling in interview-based research (Robinson, 2014), and we adopted a purposive approach. We set the following three standards to target potential interviewees: (1) the respondent is expected to be experienced in construction project management; (2) the samples are expected to cover as many application fields as possible to represent diverse construction project practices; (3) the respondent is expected to communicate voluntarily and actively. Finally, we set the bottom line of “years of management” at 5 years for the interviewee and recruited them on Zhulong BBS, China’s most influential online construction and architectural portal and community. In China, many CPMs with diverse ages, levels of experience, and working fields communicate together on this forum. A list of 100 active users (who posts and replies on the forum actively) tagged with “project manager” was developed. Besides, it is noted that we targeted potential interviewees with the purpose, seeking variations in the application field to ensure an appropriate mix of project managers (Dillon and Taylor, 2015). We sent them BBS messages to introduce our research background and purpose and clarified our requirement that the years in a managerial position should be 5 years at least. If the recipients meet our needs and are willing to be an interviewee, they could reply to us for involvement. After 2-week recruiting, we got 35 CPMs who were interested in and committed to joining the research.

The concept of data saturation, which is a point at which no new information or themes are observed in the data from the completion of an additional interview, is a helpful mark in deciding sample size in qualitative research (Boddy, 2016). Creswell and Poth (2016) suggested that sample sizes of 20–30 are typical in GTM. Following this recommendation, this paper found evident saturation at the eighteenth and definitely evident saturation at the 20-s interviewee, so we stopped the interview at the twenty-fourth interviewee. Table 1 shows the information of the participants.

**TABLE 1:** Information of participants included in the interview process.

	Age	Years of management	Nature of the corporate	Domain	The latest project location	Gender
A	56	19	SO	Hydraulic engineering	Tianjin	Male
B	36	7	SO	Highway	Nanjing	Male
C	33	5	SO	Building	Shandong	Male
D	41	8	SO	Railway	Shanxi	Male
E	44	10	SO	Municipal engineering	Shanghai	Male
F	35	6	P	Building	Chongqing	Male
G	41	9	SO	Municipal engineering	Sichuan	Male
H	42	8	SO	Building	Beijing	Female
I	37	12	SO	Airport engineering	Beijing	Male
J	35	7	SO	Highway	Republic of the Congo	Male
K	52	20	SO	Railway	Anhui	Male
L	43	10	SO	Railway	Henan	Male
M	48	16	P	Building	Beijing	Male
N	39	7	SO	Highway	Hubei	Male
O	36	5	SO	Municipal engineering	Hebei	Male
P	47	15	SO	Harbor and waterway engineering	Fujian	Male
Q	41	11	SO	Hydraulic engineering	Shandong	Male
R	39	7	SO	Railway	Yunnan	Male
S	36	6	SO	Municipal engineering	Beijing	Male
T	53	20	SO	Highway	Shanghai	Male
U	44	13	SO	Airport engineering	Shandong	Male
V	41	12	SO	Highway	Shannxi	Male
W	37	6	SO	Railway	Hebei	Male
X	38	8	P	Building	Shanghai	Male

SO, state-owned corporate; P, private corporate.

The age of participants ranges from 33 to 56. The average tenure of the managerial position is 10.3 years, with a range of 5 to 20. The participants come from state-owned (87.5 percent) and private (12.5 percent) corporate, which reflects the dominance of state-owned enterprises in the Chinese construction industry (Wang et al., 2006). Participants’ professional domain spreads from hydraulic, building, railway, highway, municipal, airport, harbor, and waterway engineering. According to the Chinese classification of construction application fields, we cover all fields except three: communication and broadcasting engineering, mining engineering, and electromechanical engineering, so we justify the variation of participants’ working experience ensures the mix of CPM samples. The latest projects the recruited CPMs managed were located in 15 Chinese provinces (or centrally administered municipalities) and one overseas country. Only one of the 24 participants is female, reflecting the male dominance of the construction industry in China (Kim, 2015).

### Process of interviewing

We collected primary data by conducting face-to-face or telephone semi-structured interviews. All participants were informed about the research background and purpose, their freedom to opt out, and the right of anonymity before the interview. The authors also provided participants a one-page document before interviewing, including the following definition of EI: “the ability to perceive emotion, use emotion to facilitate thought, understand emotion, and manage emotion” (Rosete and Ciarrochi, 2005) and explanations of each dimension. This definition is consistent with the core idea of ability EI and is concise for reading. As we expected, all participants appeared familiar with the EI concept and its utility as a management tool. The project success concept was not provided as a specific definition; we presented the project success standard proposed by Chan et al. (2004) instead. The current study expected and believed that an experienced CPM can combine various factors to understand the profile of project success.

The overarching questions guiding the interview are RQ1 and RQ2 we set before. Interviewees were elicited to elaborate on specific conditions and contexts where they use EI to manage a project. The author set three questions on this aspect at the beginning of the interview. We hope these questions will help CPM recall as many EI experiences as possible. Then, two questions were used to ask participants to describe the mechanisms through which their EI may influence project success from their perspective as a leader. According to Chinese communication habits, we set one question about challenges in using EI in the end and hope that can play a supplementary role for our research. The interview protocol is provided in the Supplementary material.

The two authors conducted all interview cases, and one Ph.D. student was responsible for the interview process and was present throughout every interview. Each interview lasted between 40 and 60 min, depending on the participant understands of EI and their management experience, and all CPMs agreed that their interviews be recorded. Notably, the interview questions we reported in this paper are translated from the original Mandarin edition, and the following quote in the next section are also translated from Mandarin. We employed an extra interpreter who is certificated by Test for English Majors-Band 8 (the highest level certificate for English in China) during the translation process to ensure translation accuracy. This interpreter is a research fellow from the second author’s institution.

### Data analysis

To avoid misunderstanding and memory bias caused by time passing, every interviewee’s recording was transferred to a transcript within 2 days after the interview. The first author and the Ph.D. student who observed all interviews transcribed the data. We divided every transcript data into two parts, first is the sense-making part, which includes CPM’s views about their EI based on the Chinese construction industry and scenario descriptions about the utility of EI in management. We conclude some representative points of view about EI from the first part data and present them in the next section. The second part is mechanism-related data, and we followed Glaser’s coding process to develop the framework in this part. The two authors conducted the grounded theory analysis. First, the two authors coded transcripts, respectively. After the open coding process, two coders compared and discussed their works and agreed on each code’s concept and theme name. Then, we move to selective coding. In this process, the two coders concentrated more on the core categories they found in open selection, and finally, in the theoretical coding process, we built the core mechanism. Besides, the coders used memos as assistants throughout the GTM process. Memos are considered “the bedrock” of theory generation and tend to be free-flowing ideas of the researcher. The coders wrote memos when they had ideas about the emerging codes and their relationships.

#### Open coding

Open coding is an iterative process of conceptualization, categorization, and comparison. In the open coding process, coders need to pay adequate attention to the emerging data and keep an open mind to the interviewee’s concerns instead of the researcher’s (Charmaz, 1995). We analyzed original transcripts literally, line by line, and coded the data in as many ways as possible. During open coding, we used the constant comparison method to group codes to produce a higher level of abstraction, called categories. In analyzing the interview transcripts, we began to abstract some key or main points; then, we assigned a code—2 or 3 words that summarize the key point—to each key point (Hoda et al., 2012). To present the author’s work on open coding, an example is shown as follows:

##### Interview quotation

“… *I usually treat the team members as my younger brothers and take care of them from both work and life aspects. When I treat them like this, I believe they will know my leading style and implication: I wish they would get along like families. And then they may be more united consciously as I wish*… *you know, it is my team, and they need to guess what I am thinking and behave as I wish*… *and my EI is important in this implication process.”*

##### Key point

Treat the team members as my younger brothers and give them support from both work and life aspects.

##### Codes

Be united like families, perceived supervisor’s caring.

The completion of open coding is marked by the emergence of the core category (Glaser, 2005). So, we stopped the open coding process when we obtained preliminary concepts and core categories that we think enough to explain the relationship between CPM’s EI and the project process. We collected 89 codes that emerged from interview data directly and then abstracted them to 23 preliminary categories and seven core categories in open coding. The seven core categories include positive team atmosphere, shared vision, team cohesion, organizational citizenship behavior directed at the organization (OCB-O), perceived supervisor support, trust in leader, and (team member’s) psychological and emotional health. The emergence of core categories (abstracted from preliminary categories) and their contents are shown in Table 2.

**TABLE 2:** The emergence of core categories.

	Core categories	Preliminary categories	Contents
Team-level	Positive team atmosphere	Positive atmosphere, openness in the team, favorable environment, benign atmosphere	Atmosphere (or environment, climate) with diverse positive elements that are beneficial to teamwork.
	Shared vision	Shared vision, common goal, blueprint, common expectation	A shared vision (or goal) that is recognized by all team members.
	Team cohesion	Team cohesion, be united like families, internal solidarity	Internal power that unites the team together.
Individual-level	OCB-O	Subordinate’s job dedication, subordinate’s conscientiousness, subordinate’s obedience to leader	Organizational citizenship behavior directed at the organization.
	Perceived supervisor support	Perceived caring in subordinate’s life and work, perceived emotional comfort, perceived moral encouragement	Team member’s perceived caring and encouragement in both life and work aspects from CPM.
	Trust in leader	Trust in leader, willing to follow manager’s guidance and instructions	Team member’s emotion and cognition trust to CPM.
	Psychological and emotional health	Self-confidence, stress, upset, self-accusation	Team member’s positive or negative psychological and emotional condition.

Some concepts repeat in two columns of the table, such as “shared vision” and “trust in leader,” because they belong to both two levels (preliminary categories and core categories).

#### Selective coding

Following Glaser’s views, we treat selective coding as a transformation from “running the data” open to delimiting the coding process around a core category (Walker and Myrick, 2006). In this process, we concentrate only on the core categories that emerged in the open coding process. Since we used coding results to guide further data collection, when we found definitely evident information saturation in the 20-s interviewee, coding of the following two interviews (the twenty-third and twenty-fourth interviewees) directly started from selective coding. In the selective process, seven core categories were classified into two levels: individual or team level.

#### Theoretical coding

Theoretical coding is the process of using theory to “conceptualize how the substantive codes may relate to each other as hypotheses to be integrated into a theory.” Although this process is not necessary (Glaser, 2005), the core category that emerged naturally drove us to this process. We present the results of theoretical coding by writing up three propositions in the next section.

## Results

### Sense-making part: Feeling about emotional intelligence and application scenarios

#### Familiar with emotional intelligence and getting emotional intelligence promotion by self-learning

Results show that the whole concept of EI was not difficult to understand for Chinese CPMs as expected. Benefiting from the popularity of Daniel Goleman’s work, the EI concept is broadly spread and understood in China. Taking the CPM-D’s description as an example:

*“I think it is a common-sense knowledge, and I am quite familiar with it. Not just in management, this concept is widespread in our whole social life.”*

We find that the above views are highly consistent in interview data. Almost all interviewed CPMs were confident in understanding the EI concept and agreed that leaders’ EI is important in project management. This consensus comes from two aspects.

First, Chinese society is built on human affect and relationships. The interviewees believed that the work relationships must be handled properly, and managing emotion is critical in maintaining relationships. CPMs emphasized that their job is to “work with people” (CPM-A) rather than provide technical contributions.

*“…later, I realized that China is a relational society where people care about their face. In most cases, affect and emotion drive the work.”* (CPM-P)

On the other hand, the CPMs suggested that the role of EI is highlighted since the lack of contract governance. CPMs indicated that in the project site, formal governance like contract governance is often lacking. When there was inadequate formal governance, relational governance had to play a prominent part. PM-U proffered a detailed elaboration on this storyline.

*“Some scholars may think that contracts, rules, and regulations are essential in project management, but on the project site, many problems were not solved by contracts and rules. Nobody does that. In our industry, almost all problems within the team are solved by the ability of the project manager. At that time, EI played its role.”*

As for EI training, organizations usually provide CPMs with training classes or large lectures that invite professions in the EI and management field. More than 80% CPMs mentioned that they had accepted related training provided by their organizations, including specialized training on EI and leadership training that includes EI knowledge. The latter is more common, as CPMs indicated. However, many CPMs who accepted EI training courses stated that this type of training was not effective enough to improve their EI level and was not practical in project management. CPM-K described the general training effect:

“… *however, to be honest, these courses had only little effect, at least for me. Although the training could help establish a systematic understanding of EI for me, they are not practical and not close to actual project management.”*

Although the training offered by organizations does not work effectively, it is noteworthy that CPMs’ EI level could get promotion in two ways: mentoring and self-learning. For the first storyline, CPM-J stated that his former manager reminded him that he “might not get very far in the construction industry” if he lacked EI. Further, CPM-K added:

*“Instead of attending courses, the management knowledge taught by my former manager in project operation is rather more useful. My EI level significantly increased from when I was new to work to when I became a manager. My former manager’s guidance was important for my styles and ways of using EI. These experiences on emotion and EI were beneficial for my following management practice.”*

An old proverb in China said: “Once a teacher and always a father figure.” The mentoring relationship from this mentoring tradition could be both formal and informal (Zhou et al., 2019). According to the collected data, we noticed that a few CPMs emphasized the effectiveness of informal mentoring. For example, as CPM-D mentioned:

*“Usually, I learn some EI skills in informal situations such as having meals. If I made some mistakes that could be avoided by using EI, such as some communication problems, my former manager usually pointed out that for me and guided me on that, and then I would not make the same mistake*…*I think it is also a special form of training, but it is more closely integrated with project practice.”*

The other way for CPMs to improve their EI level is to learn from others on their initiative. CPMs were willing to observe others in the workplace to find if others were skilled in using EI. For example, after CPM-J talked about their training organized by their company, he volunteered to speak about self-learning way:

*“Well, training opportunities were rich, but if the training were non-obligatory, I would not go because there are always many other things to do. But since I know that EI is important, I always observe emotionally intelligent others to learn from their expression of emotion and EI skills. This is a more flexible process that I can take advantage of spare time to improve my EI.”*

#### Male-dominated, but not influential in emotional intelligence management

When we asked questions about male domination in the project team, almost all CPMs acknowledged the existence of this phenomenon:

*“Even though I am a female project manager, I think this industry is still a men’s world. I only know one other female project manager like me.”* (CPM-H)

But we found that they did not think male-dominated culture is an obstacle to emotional expression. Some CPMs even appeared confused about the second question in the interview protocol about the influence of male-dominated culture. CPMs agreed that it was difficult for them to understand followers’ thinking and feeling if they “do not like to show their emotions” (CPM-X). But they believed this kind of difficulty could “happen in any industry” (CPM-W). CPM-H elaborated this view in detail:

*“Although people might think that women are more sensitive in emotions, I do not think this difference could influence project management. I believe in my team everyone could display their emotions if they want, even sometimes people hide their emotions, that is not because of their gender. I sometimes had difficulties in EI management, but it was more about their character or personality rather than gender.”*

#### Representative emotional intelligence management scenarios

We listed three representative scenarios that CPMs mentioned about EI management in Table 3. Criticizing subordinates is the most common scenario that CPMs use EI consciously. All too often, leaders tend to show criticism behavior to subordinates when they are angry or upset or fail to hold their tempers (Wang et al., 2018). However, the results of this study show that most of China’s CPMs choose not to display anger by using EI, which reflects the self-management ability of emotion. More often, given the mianzi culture (which means saving face for others and themselves) rooted in Chinese tradition (Filieri and Lin, 2017), most CPMs opt to save face for subordinates by using tender emotional expression to convey their criticism or dissatisfaction rather than be angry. For example:

**TABLE 3:** Representative scenarios in Chinese construction project manager’s (CPM’s) emotional intelligence (EI) management.

	Scenarios	Numbers of mention
1	Criticizing subordinates	16
2	Assigning temporary or extra tasks	10
3	Project launch meeting	7

*“When one of my team members made a mistake, and I needed to point it out, I used to be mad or angry and often criticized him in public. But then I started to control myself on this abreaction of emotions because I realized that this behavior is useless in a subordinate’s development and can make him lose face if I criticize him with bad emotion in front of others. And if they feel like they’ve lost face, they may be more upset and cannot work actively. Now, if I want to criticize a subordinate, I always adopt tender ways, try to be patient, and that causes good results.”* (CPM-J)

The second most frequently mentioned scenario is assigning temporary tasks. The construction project team itself is organized in a temporary environment (Wu et al., 2017), and the group can be assigned many temporary jobs caused by requirement change, design change, claim, and so on. Some CPMs demonstrated that compared to regular tasks, allocating temporary or extra tasks matters to EI.

*“It is an understandable reaction that sometimes they feel unhappy about some temporary tasks, especially when they are already busy enough. I usually pay more attention to their emotions at this time, in case that they feel stress or complaining but do not solve them timely and leads to more serious outcomes for project and themselves.”* (CPM-H)

The third frequently mentioned scenario is the project launch meeting. Seven interviewees believe it is necessary to use EI skills in this situation because it is the first formal time for them to build authority and show their working style to the whole team.

*“The first time is important, in which I need to make all things clear such as my characters, principles and some team norms. With the help of EI, I could guide members’ emotions from the start and start a good opening for the project.”* (CPM-W)

### Mechanism building part: Team level and individual level

**FIGURE 2:**
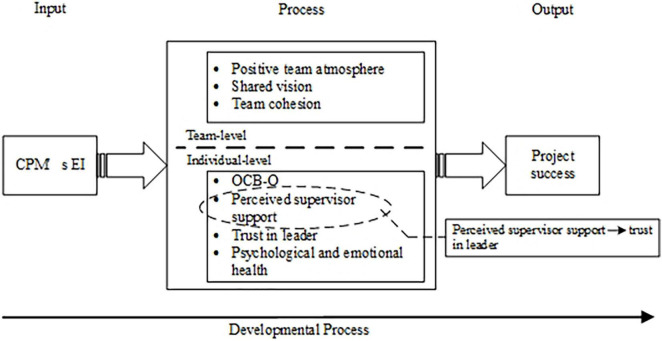
A mechanism of construction project manager’s (CPM’s) emotional intelligence (EI) on project success.

After open and selective coding, we sorted two levels of mechanism to explain the impact of CPMs’ EI on project success: the team-level mechanism and the individual-level mechanism. The theory mechanism is built based on CPMs’ *stated* opinions, and the framework is shown in Figure 2.

As a typical temporary organization context, most work in the construction industry is performed and relies on the project team (Braun et al., 2012). Recent developments in teamwork have increased the need to explore better ways to utilize teams and achieve effectiveness in the construction sector (Azmy, 2015). Views of CPM from interviews reflect the idea and spirit of team management, so we adopted the input–process–output (IPO) model in team theory to construct the influencing mechanism between CPM’s EI and project success. The IPO model was developed for researching team effectiveness by McGrath and now has been modified and extended to explain many more team issues (Mathieu et al., 2008). This model provides a framework for conceptualizing the pivotal role of the team process in mediating the conversion of inputs to outcomes. Project management researchers like Scott-Young and Samson (2008) also highlighted the need for more team process-related variables to deeply examine the relationship between the role of the project manager and project outcomes. In the IPO model, inputs describe the antecedent factors that enable and constrain team members’ interactions, which is CPM’s EI in this study. The outcome is the result of team activity and is set up as project success in the current study. Process describes members’ interactions directed toward task accomplishment. We build the mechanism based on CPMs’ *stated* opinions. As shown in Figure 2, positive team atmosphere, shared vision, and team cohesion belong to the team-level mechanism, which reflects the process variables in the sense of the whole team. The individual-level mechanism, which demonstrates the individual’s status or behaviors, includes OCB-O, perceived supervisor support, trust in leader, and psychological and emotional health.

#### Team-level mechanism

We identified three team-level influencing paths from collected data and made the following proposition:

*P1:* CPM’s EI impacts project success through a team-level mechanism, including the positive team atmosphere, shared vision, and team cohesion.

##### Positive team atmosphere

Construction project managers emphasized the overall positive tone of the team atmosphere. From the perspective of dichotomy, CPMs believed that a positive atmosphere is better than a negative atmosphere for project success, and their EI was essential in creating and maintaining this positive atmosphere.

*“One important aspect which I believe that my EI can influence is the team atmosphere. When I was an ordinary engineer before I became a project manager, my former manager told me that the atmosphere of a project team is just like the weather. He meant that if the project manager is optimistic, the project team will be positive, like a sunny day. On the contrary, if the manager is not ‘sunny’, the whole project team may suffer from rainy or snowy days, which is not good for project outcomes. In my management experience, I always try to use my EI to create that sunny atmosphere, in which all members could enjoy their work and create a good performance.”* (CPM-T)

A few CPMs talked about regulating and expressing emotion (EI ability) in creating and maintaining a positive atmosphere. CPMs began with the emotional expression of themselves, and then, by appropriate emotional contagion process, they held the whole team in such a favorable atmosphere.

*“I usually try to keep myself in a positive mood or emotion firstly, and then I will show this positive emotion to the team. It seems like that I created a positive environment from me as an original point and spread to the whole team.”* (CPM-C)

##### Shared vision

Interview results also show that emotionally intelligent CPMs could foster the acceptance of the team’s shared goals or vision, which has been argued to be critical in achieving high efficiency and optimal performance in construction projects (Zuo et al., 2018).

*“As you have known, the working and living condition in the construction site is very hard, and I need to establish something that the whole team can imagine. I have to use my emotion conveying to the team a common goal to fight for, and it not must be the project goal, some goals such as organizational reward are often better than objective project task standard.”* (CPM-I)

Construction project managers articulated a compelling vision of the project’s future by using emotions, such as “excited” (CPM-B, Q), “persuasive” (CPM-B, E, F), and “inspiring” (CPM-B, I) in describing the vision. By managing others’ emotions, CPM made the shared vision clear and guidable for the team.

*“For example, some gestures and language skills to ensure the team goals were inspiring enough and conveyed effectively. I think those skills may attribute to my EI.”* (CPM-X)

Further, EI helped CPMs judge the team members’ acceptance of the vision by assessing team members’ emotions. When members showed “carelessness”, CPM would “pay extra attention” (CPM-M) to them.

##### Team cohesion

The cohesion concept emerged directly in interview data since it is a daily word in China society, and it is almost a consensus that cohesion can improve team output. This study found that CPM’s EI was used to cultivate team cohesion. In the leader–team relationship, the formatting process of cohesion starts with the “unilateral behavior influence” (CPM-O) from CPM to team members. CPM-I described that he often presented behaviors that implied cohesiveness (such as “invite subordinates to dinner together”) with appropriate emotional expression. Other examples like CPM-G illustrated that he usually showed “expecting” or “excited” before participating in group activities. From the CPMs’ view, team members could recognize their intention and give feedback; i.e., they would be more willing to engage with the group under the guidance of the CPM.

On the other hand, EI plays a crucial role in triggering team members’ unity by CPM’s relationship management ability. CPMs use conscious emotion guidance, and then, individuals within the project team will spontaneously express cohesion-related behavior gradually, which further contributes to project success. We quote CPM-E to show this process:

*“I may treat team members as my brothers or sisters, be all of one mind. When I treat the team in this way, I believe they will know my management style and my implication: I wish the team uniting like a big family, and they may be more united consciously to me and each other. Because you know, it is my team, and they need to guess what I am thinking and follow my rhythm.”*

#### Individual-level mechanism

We identified four individual-level influencing paths and made the following proposition:

*P2*: CPM’s EI impacts project success through an individual-level mechanism, including OCB-O, perceived supervisor support, trust in leader, and subordinate’s psychological and emotional health.

##### Organizational citizenship behavior directed at the organization

When the preliminary categories “subordinate’s job dedication,” “conscientiousness,” and “obedience” emerged one by one, we found that all these concepts belong to a common higher-level theme: OCB-O. OCB is defined as “individual behavior that is discretionary, not directly or explicitly recognized by the formal reward system, and that in the aggregate promotes the effective functioning of the organization” (Organ, 1988). OCB-O is OCB directed at the organization, such as loyalty, obedience, participation, job dedication, conscientiousness, civic virtue (Spitzmuller et al., 2008). The approach of EI leaders influencing subordinates’ OCB-O may attribute to their ability to integrate emotions in themselves and others to devise effective strategies. For example, when CPM-M expressed emotions such as “serious” or stating tasks authoritatively, team members may be influenced and tend to show obedience behavior.

*“Project management in the construction site cannot reach a high level of detail sometimes. So it will be easier if people feel respect or a little fear for me. I need to build my authority by expressing some specific emotion, such as be serious, or angry, and so on.”*

Other positive emotions, such as being “mild” (CPM-D, N, O), “patient” (CPM-A, E), and “enthusiastic” (CPM-K, U), can strengthen subordinates’ job dedication and conscientiousness behaviors. For example, CPM-E mentioned that:

*“I think individual performance is essential for achieving project success. I believe people in the team are qualified enough for project work since they have passed the recruiting department’s necessary examination in many aspects, so their technical profession was not what I was concerned about. Their work conscientiousness or involvement is more important. Once they recognize that they are part of the team, they will have more conscientiousness and work more proactively. So in the communication between us, I always show positive emotion to make them realize the value of their work for the whole team, with my patience, to influence them.”*

##### Perceived supervisor support

Some CPMs mentioned their support-offering behaviors and attached importance to team members’ perception of that support. They believed that leader’s support is beneficial for team members’ performance and project success.

“Emotional comfort” (CPM-C, F, K, L) is an outstanding aspect of CPM’s support, and it is directly related to CPM’s EI. In the process of offering emotional comfort, CPM needs to integrate all EI skills. Some CPMs considered it “challenging work”. We quote CPM-L’s statement:

*“Young people liked to talk with me about their work stress or other things about their life. And I could give them guidable feedback or something like emotional comfort. But it’s not that easy to comfort someone, especially when I’m his leader.”*

Construction project managers utilized EI in supporting subordinates’ work progress. Emotion controlling left subordinates the impression that the leader is “professional” and “made them willing to accept supervisor’s work guidance” (CPM-V).

Supporting the “daily life of team members” is also emphasized by many project managers. CPMs cared about followers’ “health” (11 times mentioned), “families” (seven times mentioned), and “financial situation” (five times mentioned). CPM-N noted that when team members perceived that the leader cared about their life, they were generally “more engaged in their work.”

##### Trust in leader

Interviewee believed that their EI could strengthen subordinates’ trust and further achieve success. Emotion such as being “self-confident” (CPM-B, T, V) or “calm” (CPM-B, I) expressed by CPM could create a sense of security to team members and lead to their trust in CPM. As CPM-F mentioned:

*“Whatever emergency happens, if they see that I am not anxious and I am in control, they would believe that I can handle this condition. Then they will try their best too, for the project completion.”*

##### Perceived supervisor support and trust in leader

The relationship between the concept “perceived supervisor support” and “trust in leader” emerged from interview data, and this is the only emerged concept dyad. We found that almost all CPMs who mentioned the trust in manager concept pointed to the perceived supervisor support concept. CPMs believed their EI could promote followers’ perceived support and then improve trust in the leader. Representative statements are as follows:

*“Interpersonal relationship is mutual, and if they realize I am not only a supervisor but a friend or family when they perceive my care to them, we will build emotional connection, and they will trust me.”* (CPM-L)

##### Psychological and emotional health

We summed up a series of concepts (including self-confidence, stress, upset, self-condemned, etc.) together as one core category: subordinate’s psychological and emotional health. Notably, for positive variables such as self-confidence, CPM’s EI could facilitate them and play a positive role in achieving project success. On the other hand, for negative psychological and emotional conditions such as stress, EI could buffer their harmful effects on project success reversely.

A few CPMs said they were sensitive about followers’ “nervous” and “frustrated” emotions (CPM-H, I, N). We believed that these CPMs were good at identifying emotions in team members. We quote some transcripts on different psychological and emotional health concepts here:

*“I suppose that my EI is helpful for followers’ confidence building. There are many 90s in the project team now, and when they involve in their first project, they feel unfamiliar with site practice and have little self-confidence. At that time, their first project manager in their career is important for them. I am always patient enough to take care of those freshmen, to recognize and feel their depression and express my praise and appreciation.”* (CPM-G, self-confidence)

*“If you show emotions such as “worried” when allocating tasks, you may undermine their confidence.”* (CPM-H, self-confidence)

*“By helping them stop feeling guilty about their past mistakes, they can perk up and contribute more to the future of the group.”* (CPM-F, self-condemned)

*“If someone had a high level of stress for a long time, his work effectiveness would go down. Sometimes they hide this psychological stress, and I may ignore this secret worry if I do not have enough EI. On the contrary, once I am aware of their stress, I can take action to help them.”* (CPM-Q, stress)

### A mechanism that develops over time

Except for the mediating mechanism, we also identified and defined “time developmental” as a critical property of this model and proposed:

*P3:* The mechanism develops over time; CPMs’ EI’s influence on project success is a slowly but gradual accumulating process.

We are glad to see this emerged property because this time character is in keeping with the extant IPO team theory (McGrath, 1964). This time-developmental character means the effect of this mediating mechanism does not work immediately. CPM-M elaborated this point in detail:

*“My EI did work as an important factor for project success. However, this process took a long time. Compared to other direct behaviors I behave, EI is more obscure and needs more understanding from followers. If we are working on a large and complex project that may take five or ten years, the influence of my EI may work on “this” project. Or, if the project we are fighting for is limited to a short time, the contribution of my EI may manifest in the “next” project or the “after next” project. That is to say, my efforts on EI management may not be able to contribute to the success of the current project.”*

In conclusion, project success is differentially influenced by various factors influenced by CPMs EI (team level or individual level) as the team matures over time. We refer to the developmental model to depict this time character (Mathieu et al., 2008). The solid line running at the bottom of Figure 2 shows that the developmental processes unfold over time.

## Discussion

### Chinese construction project manager’s views on emotional intelligence: Critical ability in guanxi culture

We argue that although the male-dominated culture is widely present in the Chinese construction industry, EI is not an unacceptable concept in Chinese society and CPMs embrace this ability or leadership skills in project management. This phenomenon is different from Lindebaum and Cassell’s (2012) result and may be explained by Chinese traditional culture.

Guanxi culture is rooted in traditional Chinese relationalism, which describes a body of work conceptualizing people’s social existence and connectivity, not as individual beings but as relational beings (Xu and Dellaportas, 2021). Everyone in Chinese society is born not as an individual but as someone’s son or daughter, brother or sister (Fan, 2010). In other words, the individual role is attached to his/her social status and position in a specific relationship. Compared to the Western society where CPMs may think avoiding emotion helps them keep professional, Chinese CPMs’ leadership is embedded in their relationships where an individual’s status is defined. CPM refers to *a leader in a team*. CPMs naturally care about their guanxi with everyone on the team, driven by conventional relationalism. Therefore, they want to create a positive atmosphere in the group, save subordinates’ faces, focus on whether team members trust them, etc. The perceiving emotion, using, understanding, and managing emotion abilities are essential that CPMs naturally use to manage relationships. CPMs train their EI by self-learning is also explained by guanxi culture since they need to improve EI while the organization does not provide enough support.

Conclusively, in the Chinese construction industry, the emotion avoidance phenomenon under male-dominated culture does not present since a more powerful cultural incentive pushes CPMs to show emotion, use EI, and improve EI.

### Mechanism building: Management practice to theory

Based on existing theories, we now discuss the mediating mechanism established in this paper.

#### Team-level mechanism

We found that CPMs believe that EI could influence project success by creating a positive project atmosphere. According to extant theory and literature, leaders with a high level of EI could leave a good impression on others, which is beneficial to improve the team’s internal positive emotion (Schlechter and Strauss, 2008; Waldron, 2017). EI leaders tend to create an effective atmosphere where team members could live in harmony and communicate openly, cognitively interact more with each other, and intrinsically enjoy their work (Wei et al., 2016; Zhang et al., 2018). The effective atmosphere mentioned above shows a positive tone consistent with the positive atmosphere emphasized in the current study. Secondly, existing studies support the role of a leader’s EI in promoting shared vision. The emotion perception and understanding abilities enable individuals to perceive the development demands of others, clarify the shared vision, and call on others’ effort for it by their appeal and influence (Sunindijo and Zou, 2013). EI leaders with empathy nature can perceive the acceptance and recognition of subordinates to team vision effectively and adjust their behavior and attitude toward subordinates based on their feedback (Pinos et al., 2013). Empirical evidence in the organizational context shows that EI leader creates a shared vision among the various stakeholders within the decision-making team and gets others excited about the vision by using positive emotional contagion (Boyatzis and Soler, 2012). Therefore, we could see the consistency in some existing researches and this study, though they are rooted in different team contexts. For the team cohesion concept, according to Mohammad et al. (2014), EI leaders can subtly influence employees’ practices at work, such as building team cohesion. The positive emotion from CPM is usually a signal, which means that the leader is satisfied with the existing task performance. This positive signal can make team members feel comfortable with the current communication and cooperation process in work and enhance team cohesion (Chan and Mallett, 2011). From another perspective, some research supports that leaders in institutional collectivism culture tend to use their EI to build a collective identity to cultivate the team’s loyalty and cohesion (Miao et al., 2016). China, influenced by Confucian culture, is a significant collectivist nation. Some existing research supports that EI positively impacts project team performance and cohesiveness; however, parts concentrate on team EI instead of project manager’s EI (e.g., Quoidbach and Hansenne, 2009). Therefore, we treat our finding on team cohesion as a supplement for extant empirical literature.

#### Individual-level mechanism

Interviewed CPMs agreed that OCB-O is crucial for project success, keeping with extant literature such as Braun et al. (2013) and Guo et al. (2016). A meta-analysis conducted by Miao et al. (2018) shows that leaders’ EI demonstrates incremental validity and relative weight in predicting subordinates’ OCB even after controlling for personality factors and cognitive ability. People with good emotion evaluating and expressing abilities usually show a high level of empathy, respect, and personalized consideration (Salovey and Mayer, 1990). These characteristics help leaders influence their subordinates’ values and aspirations and do more work than expected by stimulating their higher-level needs (Bass and Avolio, 1990; Yukl, 2002). CPM’s respect for the team can create a pride feeling among team members, leading to more extraordinary efforts and behaviors of taking on extra work (Irshad and Hashmi, 2014). From the cultural perspective, the Chinese construction industry is in a culture of high power distance (Tsai and Chi, 2009), and subordinates’ obedience is easier to be influenced by leaders’ EI in this kind of culture (Miao et al., 2018). Therefore, we could see some consistency between our findings and existing research. Secondly, the PSS path we built conform to the existing argument that EI leader tends to perform care and support, individualized consideration, and respect to followers, this property roots in and reflects the EI nature (Salovey and Mayer, 1990). EI leaders, just like transformational leaders, are often seen as powerful examples for followers, and when such role models show personal support to subordinates, team members are more likely to perceive and accept such care and support (Eisenberger et al., 2016). Bro et al. (2017) suggested that employees, who perceive that their leaders care for them, are motivated to do more than they are expected to do. Therefore, although few empirical studies directly investigated the relationship between CPM’s EI and perceived supervisor support, we could find related implications from prior studies. Thirdly, social exchange theory can help explain the mediating role of trust in the leader concept and the relationship between PSS and trust in leader. As we mentioned above, EI leaders usually show a high level of respect for their followers. EI assists leaders in understanding followers’ situations and better recognizing those situations where additional supports are needed (Clarke and Mahadi, 2017). To feedback on the leader’s care and respect, subordinates tend to show high affect-based trust in their leader (Prabhakar, 2008). Some others said that when followers perceive and experience a leader’s support, they have the recognition of being valued in this leader–member relationship, which further pushes them to reciprocate in terms of their emotional attachment, thereby developing trust in their leader (Colquitt et al., 2012; Zhu and Akhtar, 2014). Some empirical studies support that a leader’s ability to use emotion, understand emotion, and regulate emotion is positively related to an employees’ trust in their leader in an organizational context (Sitter, 2004). Trust is also found to be a predictor of project performance and project effectiveness (Rezvani et al., 2016). Finally, for the psychological and emotional health concept, existing research has demonstrated that leaders’ EI influences employees’ attitudes (Trejo, 2014), and EI leaders could bring followers about motivation effectively (Èiutienë et al., 2019). In the workplace environment, leaders become mood managers of their employees by helping their employees overcome the mood-damaging effects of negative events and to help to curb their stresses (Humphrey, 2015). Emotionally intelligent CPMs create emotional synchronization (or resonance) by showing confident and enthusiastic emotional displays to enhance their employees’ positive mind conditions and reduce their negative mind conditions (Humphrey et al., 2008). Just as Podsakoff et al. (1996) suggested, leaders need to develop an understanding of the variables that influence subordinates’ mental conditions and develop a clear understanding of how to affect these variables. This study summed these variables together to a vital influence path in the relationship between CPM’s EI and project success.

### Time character: Manifestation of team developmental process

Team developmental processes unfold over time as teams mature (Mathieu et al., 2008), and the time character we found conforms to the inherent cyclical nature of team functioning (Ilgen et al., 2005). Project managers use their leadership to transform loose teams into effective teams throughout the project (Sheard and Kakabadse, 2002), and EI’s influence will be gradually profound with the team’s development. Specifically, in the team’s early days, CPMs’ decision-making is more likely to be affected by the project owner’s suggestion since the project objective is not going into detail (Senaratne and Samaraweera, 2015). As the team evolves and leader–member relationships develop, CPM’s EI begins to help team members solve interpersonal problems to achieve team goals (Senaratne and Samaraweera, 2015), and the sensitivity and empathy traits of CPM’s EI are therefore more powerful. The time character we found is a novelty phenomenon in the project management field but conforms to classical team developmental theory, which says construction project team outcomes are likely to be influenced by their progress over time.

## Conclusion

### Contributions

The contributions of this qualitative study are of major significance to the field of project management. *Firstly*, this study reveals the true feelings and thoughts of Chinese CPMs on EI and finds the inapplicability of the conclusion based on developed countries (e.g., Lindebaum and Cassell, 2012; Lindebaum and Jordan, 2012). The results show that CPMs embrace their EI ability in project management under the Chinese guanxi culture rather than avoiding using it. Although the concept of EI is well-known in China, few qualitative studies comprehensively describe CPM’s views and application status of EI in the Chinese construction industry. This study fills the gap in this field. *Secondly*, this study answers the calls for exploring variables that potentially mediate the EI–project success relationship (Müller and Jugdev, 2012) and finds new possibilities to analyze CPM’s EI toward project success. These findings broaden a new vision to explain CPM’s EI’s influence on project success from the team process’s perspective. This theory model could help realize the power of CPM’s EI on the project team and team members. The mechanism framework we built is notable and waiting to be confirmed by quantitative research. *Thirdly*, this research is the first one that points out the role of “time” in the influence process from CPM’s EI to project success and combines CPM’s EI with existing team theory. This result implies that it is necessary to conduct empirical research using longitude data in EI research within the project context.

### Limitations and implications

Several limitations of this study should be noted, and they raise questions that future research could explore deeply. First, this current study interviewed project manager samples, which may cause social desirability bias in describing the influence process (Fisher, 1993). The social desirability bias may lead to CPMs reporting those influence paths they *think* are desirable to identify rather than the actual mechanism that works in the project team. That is why we emphasize that the conclusions of this work are based on CPMs’ *stated* opinions in the different places of the paper. For future research, we can go further and collect information from team members to explore the mechanism that project managers *show* rather than what they *think or believe* in the management process. Another common limitation of applying GT is that GTM lacks data validation, unlike most other “scientific methods.” Glaser clearly states that the focus of GT is the *generation* of theory, and validation may be undertaken by other researchers using different methods. We, however, found that several activities during GTM research can help evaluate and validate the emerging results of a GTM study. One way is to see whether the result also explains and fits the experiences of different practitioners not involved in theory generation. Specifically, we presented the emerging results to some other construction practitioners from the member companies of the China Construction Industry Association (CCIA). Many member companies cooperate with the second authors’ university for the registered constructor education training program permitted by CCIA. These practitioners helped the validation of our findings, and we believe our results are representative. Finally, an inherent limitation originating from GTM is that the resulting theory can only explain the specific contexts explored in our study—the Chinese construction context. In fact, we started from this context and tried to describe situations in this context, but we cannot test the external validity of this study. Therefore, we call for more investigations based on diverse geographic, economic, cultural, and legal backgrounds. Besides, the result of the current research is a theoretical mechanism, which needs to be tested and refined through other empirical or quantified research.

For management practices of the construction industry, this research provides a theoretical basis for Chinese construction enterprises to carry out professional EI training. Generally, leadership training in Chinese construction companies has been extensive (Jie, 2019); however, corporate EI training that solid, evidence-based guidance for assessing and training emotional skills in organizations remains scarce (Lopes, 2016). Practical EI training to improve CPM’s EI level exerts lasting and long-term effects to project team members. Therefore, we argue that construction enterprises should establish special EI training close to project site management for CPMs. For example, the representative EI management scenarios we found in this study can be set as scene exercises to make CPMs familiar with the utility of EI. Moreover, corporates should help CPMs understand the role of EI in team management, such as EI’s impacts on the variables included in the model we built. In addition, when companies hire and select project managers for projects, a candidate’s EI level seems to be valuable for decision-making.

## Data availability statement

The original contributions presented in this study are included in the article/Supplementary material, further inquiries can be directed to the corresponding author.

## Author contributions

SH designed the research question, guided the research design, and was involved in the interview. QZ designed the interview process, was involved in the interview, and drafted the manuscript. Both authors coordinated and contributed to the manuscript and approved the submitted version.
