# Supplementary material for: Developing a mechanism of construction project manager’s emotional intelligence on project success: A grounded theory research based in China
Source: Front Psychol. 2022 Sep 26;13:693516. doi: 10.3389/fpsyg.2022.693516 (PMC9549140; doi:10.3389/fpsyg.2022.693516)
Supplement: Supplementary file 1 [file Table_1.DOCX]

# Interview Protocol

1. Are you familiar with the definition of EI and have you accepted related training since or before you promoted to project manager?

2. Do you think Chinese construction industry is male-dominated and does this phenomenon impact the display of emotion in workplace?

3. Have you used some EI skills consciously for managing project, or even at that past time you did not use it consciously but when you see this question right now you can recall and realize some examples of EI using? (Think about a recent project and give some specific examples)

4. What aspects do you think the project manager’s EI may influence?

5. Do you think project manager’s EI is important to project success, and talk about why?

6. Think about some conditions where you felt you met with a lot of problems, or one you found particularly challenging in using EI to manage project.

(*Note.* Above questions are translated from Chinese)
